# Knowledge, practice and attitudes of healthcare students to sepsis management in Jamaica

**DOI:** 10.1186/s12909-025-07122-w

**Published:** 2025-04-17

**Authors:** Karen J. Roye-Green, Ivan Vickers, Sharon Priestley, Jerome Walker, Rohan Willis

**Affiliations:** 1 Medical Branch, Internal Medicine Rheumatology Division, University of Texas, Galveston, USA; 2 Department of Microbiology, Faculty of Medical Sciences, University of the West Indies, Mona, Kingston, Jamaica; 3 Department of Sociology, Psychology and Social Work, Faculty of Social Sciences, University of the West Indies,Mona, Kingston, Jamaica

**Keywords:** Sepsis care bundles, Sepsis education, Healthcare students, Sepsis, Knowledge, Attitudes, Practice, Management, Jamaica

## Abstract

**Background:**

Sepsis is a medical emergency requiring timely management and available global evidence suggests that healthcare workers and students are poorly prepared to effectively diagnose and treat such patients. This study evaluates the inter-relationship of healthcare students’ attitudes towards, knowledge of and practice of sepsis management as they progress through training in Jamaica.

**Methods:**

A prospective cross-sectional survey using an anonymous self-administered questionnaire with convenience sampling was performed among healthcare students at all levels of training. All available medical and nursing students from the major public medical and nursing schools in the Kingston Metropolitan Area were included in the study. The questionnaire was composed of 25 items covering aspects of the knowledge, attitudes, and practice of sepsis management.

**Results:**

The study population consisted of 292 respondents; 210 medical and 82 nursing students. The need for fluid resuscitation before ICU admission (72.6%) was the practice question that was correctly identified by the majority of students. Most of the remaining items were correctly identified by approximately half of the students including signs of sepsis such as altered mental state (56.1%), low systolic blood pressure (53.7%) and tachypnea (50.6%). In contrast, very few students could identify the signs that indicated the presence of septic shock such as high serum lactate and the need for vasopressors and only 7% of students knew the correct annual sepsis mortality rate. Nursing students had higher overall mean correct knowledge and correct practice scores compared to medical students and lower incorrect practice scores, although there was no difference in incorrect knowledge scores between the 2 respondent groups. A subgroup analysis of students in their final stage of training revealed a more comparable performance of the 2 student groups, highlighting the improved performance by both nursing and medical students who received either formal sepsis training or were in the late stage of training. Jamaican healthcare students agree that more training on sepsis is needed (98.3%) and that sepsis care bundles should be implemented during their training courses (94.2%).

**Conclusions:**

This study revealed differences in the healthcare students’ attitudes, knowledge of and practice of sepsis in Jamaica. There is the need for training on sepsis and implementation of sepsis care bundles.

**Supplementary Information:**

The online version contains supplementary material available at 10.1186/s12909-025-07122-w.

## Background

Sepsis is a life-threatening organ dysfunction caused by a dysregulated host response to infection [1]. Sepsis syndrome is associated with significant morbidity and mortality. It affects 48.9 million individuals with a resultant 11 million sepsis- related deaths worldwide based on 2017 estimates [2]. Sepsis is the most common cause of in-hospital deaths with over $24 billion USD annual cost in the United States (US). While the true incidence of sepsis is unknown, it is estimated to kill between one in three and one in six affected persons [3–5] or one in four on average [4]. The continued trend of persisting high prevalence of sepsis despite improvements in vaccines, antibiotic therapy and acute care is due to an increase in risk factors such as an aging population, invasive procedures, chemotherapy, malnutrition and immunosuppression [6–8].

Sepsis is a medical emergency that requires early diagnosis and treatment to improve patient outcomes and therefore appropriate training and heightened awareness of healthcare workers is critical in this regard [9]. As such, the Surviving Sepsis Campaign (SSC) was developed to provide guidance for care of adults with sepsis or septic shock in the hospital setting. The international implementation of SSC bundles consisted of sepsis screening, performance, education and audit with feedback reflecting improvements according to current clinical practice, with the last update in 2021 [10]. The appropriate implementation and compliance with SSC bundles have been associated with decreased mortality due to sepsis; rates decreasing 0.7% for every 3 months that a hospital implemented and participated in the SSC [11]. Decreased mortality was noted following SSC implementation with specific protocols for sepsis care in New York as well as in several Intensive Care Units (ICUs) in the US, Europe and South America compared to centers that did not implement these protocols [12–15].

The management principles of sepsis care bundles include doing blood cultures prior to antibiotic administration, the use of intravenous broad-spectrum antibiotics followed by fluid resuscitation and vasopressors for hypotension when indicated, and the determination of serum lactate to guide therapy. Despite clear evidence for the efficacy of sepsis care bundle implementation in hospital systems, there is limited application of this sepsis management tool globally, particularly in developing countries [16]. Several reports indicate that healthcare workers and students are poorly prepared to effectively diagnose and manage septic patients [17–22]. A previous survey of healthcare professionals in Jamaica demonstrated clearly that more sepsis education is required and management of sepsis may benefit from implementation of SSC in Jamaican hospitals [23]. The aim of this study is to evaluate the inter-relationship of Jamaican medical and nursing students’ attitudes towards, knowledge of and practice of sepsis management as they progress through their training course in an effort to provide insight on the suitability of the SSC for Jamaican hospitals.

## Methods

### Ethical approval

Ethical approval was obtained from The University Hospital of the West Indies/The University of the West Indies (UHWI/UWI) Faculty of Medical Sciences (FMS) Ethics Committee Mona (ECP.32, 16, /17) and Ethics Committee of Ministry of Health of Jamaica (2016/31).

### Questionnaire development and administration

The data collection tool, a 25-item questionnaire, was developed by the study authors (KRG, IV) and adjusted after an initial pilot study as outlined previously [23]. The initial pilot study questionnaire draft included 20 respondents (5 doctors, 5 nurses, 5 medical students and 5 nursing students) and based on this initial validation, several changes were made to improve the content, reliability and appropriate difficulty of the instrument. A sample of the final questionnaire is provided with details of the scoring system as outlined below (Supplementary file S1). These included removing redundancies, the demographic questions (Q1-9) were adjusted to include open-ended options where appropriate to improve data capture, additional questions were included in the “Attitude” section and an option for “don’t know” was added to several questions. The first section consisted of open-ended questions on demographic data (year of training occupation, gender and healthcare institution), while the remaining 3 sections were formatted as multiple-choice questions covering attitudes, knowledge and practice according to sepsis management outlined in the 2016 International Sepsis Guidelines. It is important to note that a single question may have multiple correct and incorrect answers and so we developed independent ‘correct’ and ‘incorrect’ scores to more accurately evaluate respondents. At the time of the survey, the 2016 SSC guidelines were current and all respondents were informed that the questionnaire would assess their awareness on current sepsis practice guidelines.

We conducted a prospective, cross-sectional study using an anonymous self-administered questionnaire between June 15, 2018 and June 14, 2019. All healthcare students from public Jamaican Institutions were eligible for inclusion in the study. Convenience sampling of all available medical students at UHWI, the only public medical school, and nursing students from the 3 major public nursing schools completing training at the UHWI, Spanish Town Hospital [24], Kingston Public Hospital (KPH) or the Bustamante Children Hospital (BCH) was performed across all years of the relevant training course. The typical training course for nursing and medical students spans a total of 4 years (Y1-Y4) and 5 years (Y1-Y5), respectively. After informed consent was obtained from study participants, they completed and returned the questionnaire without an opportunity to research answers beforehand.

### Assessment of respondent attitudes towards Sepsis management

The attitudes towards sepsis management were assessed by multiple choice questions 15–17 on the questionnaire, to ascertain participants’ feelings regarding the ability of healthcare students to recognize patients most at risk for sepsis and the need for sepsis training.

### Assessment of respondent practice and knowledge of Sepsis management

These final 2 sections, sepsis knowledge (Q10-14) and practice (Q18-24), were assessed quantitatively, with each correct answer of the available multiple choice responses being allocated a value of 1 point contributing to the ‘correct knowledge’ or ‘correct practice’ score. Similarly, each incorrect answer was allocated a value of 1 point contributing to the ‘incorrect knowledge’ or ‘incorrect practice’ score. Details of the tabulation method are outlined in a previous publication [23], but briefly, 4 scores were tabulated based on the points allocated: a ‘correct knowledge’ score, a ‘correct practice’ score, an ‘incorrect knowledge’ score and an ‘incorrect practice’ score. The maximum possible value for the ‘correct practice’ score was 14 points, while the maximum possible score for the ‘correct knowledge’, ‘incorrect practice’ and ‘incorrect knowledge’ scores was 7 points each.

Mean values of each of the 4 tabulated scores were calculated for each respondent group to allow comparison and to determine predictors for higher or lower scores in each group. High correct scores indicate appropriate knowledge or practice for sepsis management while high incorrect scores indicate inappropriate knowledge or practice. A sample of the questionnaire is provided with details of the scoring system (Supplementary file S1).

### Statistical analysis

Statistical analyses were performed using the Xstat Software, Version 16.6 (Addinsoft, New York, NY). The mean and standard deviation or medians with interquartile ranges were calculated for outcome variables as appropriate. The significance of the difference of score means and comparison outcomes in respondent groups were calculated using student’s t-test and ANOVA or Mann-Whitney U and Kruskal-Wallis test as appropriate. Comparisons of outcomes across years of training in medical students according to each year group (Y1 vs. Y2 vs. Y3 vs. Y4 vs. Y5). However, due to limited numbers in each individual year group for nursing students, outcomes across years of training were compared by early training (Y2 and Y3) vs. late training (Y4). No completed questionnaires were obtained from nursing students in Y1 of training, who were not purposefully excluded, however, the sampling did not capture any of these students. A subgroup analysis comparing nursing students (Y4) and medical students (Y4 & Y5) in the late stages of clinical training was performed to minimize the impact of differences in training structures and curricula as well as imbalances in the capture of students at different stages of training.

## Results

### Respondent profile

The study population consisted of 292 students; 210 (71.9%) medical and 82 (28.1%) nursing students. At the time of this survey a total of 1535 medical students were eligible for study inclusion (270 in Y1, 315 in Y2, 350 in Y3, 300 in Y4 and 300 in Y5), so the response rate for captured medical students was 13.7%. A total of 1400 nursing students (350 total per each year across the 3 major schools) were eligible for study inclusion, so the response rate was 5.9%. Most respondents were female 240 (82%) and at the time of questionnaire administration all medical students were completing training at the UHWI, the only public medical school. Most nursing students from one of 3 major nursing schools in the Kingston Metropolitan Area were completing training at 1of 4 major hospitals, the UHWI 30 (36.6%), KPH 28 (34%), STH 10 (12.2%) or BCH 3 (3.7%) while a few others 4 (4.9%) were completing training at smaller institutions. The current year of training of medical students at the time of questionnaire administration spanned Y1 (1, 1.5%), Y2 (117, 55.7%) Y3 (23,10.9%), Y4 (50, 23.8%) and Y5 (8, 3.8%). The current year of training of nursing students spanned Y2 (4, 4.9%), Y3 (1, 1.2%) and Y4 (74, 90.2%). The year of training for medical students and nursing students was not available in 11/210 (5.2%) and 3/82 (3.7%) cases respectively.

### Assessment of knowledge

Overall, most students were able to correctly identify several signs of sepsis, including altered mental state (164, 56.1%), low systolic blood pressure (BP) (157, 53.7%) and tachypnea (148, 50.6%) as well as the correct definition for sepsis as a dysregulated host response to infection (152, 52.0%). Correct responses for defining characteristics of septic shock were less frequent such as need for vasopressors (114, 39.0%) and elevated serum lactate (59, 20.2%).

Nursing and medical students were able to correctly identify most of the signs of sepsis (Q10) and the correct definition of sepsis (Q11) with similar frequency (range 45.1–62.1%). However, nursing students were able to identify a low systolic blood pressure as an indication of sepsis more frequently than med students (63.4 vs. 50.0%, *p* = 0.039). Similarly, nursing students were more likely to correctly identify signs of septic shock (Q12), including need for vasopressors (60.9 vs. 30.4%, *p* < 0.001) and high serum lactate (41.4 vs. 11.9%, *p* < 0.001) (Table 1).

Only a few students were able to identify the correct annual mortality rate (Q13), approximately 7% in both medical and nursing students, with the majority of both groups of students (57.1–65.8%) indicating that they were unaware of the actual rate. Interestingly, while both medical and nursing students indicated incorrect responses to most factual knowledge questions with similar frequency, nursing students were more likely than medical students to incorrectly identify abnormal White Blood Cell (WBC ) (76.8% vs. 63.8%, *p* = 0.033) and bacteremia (81.7% vs. 64.2%, *p* = 0.004) as defining features of sepsis (Table 1).

**Table 1:** Assessment of sepsis management knowledge in nursing and medical students (Q10-13)

Variable\Statistic	Total *N* = 292 (%)	Med Student *N* = 210 (%)	Nurse Student *N* = 82 (%)	*p*-value	
*CORRECT ANSWERS*					
Q10– Signs of Sepsis					
*-Systolic BP of 100mmHg or less*	157(53.7)	105(50.0)	52(63.4)	***0.039***	
*-Altered Mental State*	164(56.1)	113(53.8)	51(62.1)	*0.195*	
*-Respiratory rate > 22 breaths/min*	148(50.6)	109(51.9)	39(47.5)	*0.506*	
Q11– Sepsis Definition					
*- dysregulated host response to infection*	152(52.0)	115(54.7)	37(45.1)	*0.139*	
Q12– Septic Shock Definition					
*-Hypotension &Vasopressors for MAP*	114(39.0)	64(30.4)	50(60.9)	***< 0.001***	
*- Hypotension & Serum Lactate > 2mmol/L*	59(20.2)	25(11.9)	34(41.4)	***< 0.001***	
Q13– Sepsis Annual Mortality Rate					
*− 20 to 50%*	21(7.1)	15(7.14)	6(7.31)	*0.960*	
*INCORRECT ANSWERS*					
Q10– Signs of Sepsis					
*-PaCO2 < 32mmHg*	77(26.3)	57(27.1)	20(24.3)	*0.633*	
*-Abnormal WBC < 4 or > 12 × 10* ^*3*^ */ul*	197(67.4)	134(63.8)	63(76.8)	***0.033***	
Q11– Sepsis Definition					
*-Blood Poisoning*	43(14.7)	34(16.1)	9(10.9)	*0.260*	
*-Bacteremia*	202(69.1)	135(64.2)	67(81.7)	***0.004***	
*-Allergic Reaction*	6(2.0)	5(2.4)	1(1.2)	*0.532*	
Q12– Septic Shock Definition					
*- Hypotension & cardiovascular dysfunction*	127(43.4)	96(45.7)	31(37.8)	*0.222*	
Q13– Sepsis Annual Mortality Rate					
*− 1 to 5%*	8(2.7)	7(3.33)	1(1.21)	*0.322*	
*− 10–15%*	46(15.7)	37(17.6)	9(10.9)	*0.162*	
*− 20 to 30%*	43(14.7)	31(14.7)	12(14.6)	*0.979*	
*- don’t know*	174(59.5)	120(57.1)	54(65.8)	*0.174*	

MAP: mean arterial pressure, mmHg– millimeters mercury, PaCO2: arterial partial pressure carbon dioxide, WBC– white blood cell,

### Assessment of practice

Overall, the frequency of correct responses to questions about routine practice for sepsis management was widely variable from a low of 39/292 (13.3%) to a high of 212/292 (72.6%). Correct responses were most frequently provided in response to questions relating to immediate resuscitation measures (42.4 − 69.5%) and the need for fluid resuscitation before ICU admission 212/292 (72.6%). The lowest frequency of correct responses was provided in response to questions relating to indications for prolonged antimicrobial use, especially simultaneous fungal infection 39/292 (13.3%), neutropenia 55/292 (18.8%) and undrainable infectious focus 69/292 (23.6%), as well as the optimal timing of antibiotic therapy post diagnosis 59/292 (20.2%). Incorrect responses were similarly variable, with frequencies ranging from 3/292 (1.0%) to 140/292 (47.9%) (Table 2).

When comparing nursing and medical students, correct practices were indicated more frequently by nursing students and incorrect responses by medical students with a few exceptions. In particular, correct practices related to immediate resuscitation measures such as measuring lactate levels [59/82 (71.9%) vs. 65/210 (30.9%), *p* < 0.001], obtaining blood cultures before antibiotic therapy [74/82 (90.2%) vs. 111/210 (52.8%), *p* < 0.001] and the need for broad spectrum antibiotic therapy [72/82 (87.8%) vs. 131/210 (62.3%), *p* < 0.001] were correctly indicated more frequently by nursing compared to medical students. However, while the presence of a simultaneous fungal infection was noted as requiring prolonged antimicrobial therapy by only a few of the students overall, medical students were more likely to correctly identify this indication compared to nursing students [34/210 (16.1%) vs. 5/82 (6.1%), *p* = 0.023].

**Table 2:** Assessment of sepsis management practice in nursing and medical students (Q18-24)

Variable\Statistic	Total *N* = 292 (%)	Med Student *N* = 210 (%)	Nurse Student *N* = 82 (%)	*p*-value
CORRECT ANSWERS				
Q18– Immediate Resuscitation Measures				
*Measure Lactate*	124(42.4)	65(30.9)	59(71.9)	***< 0.001***
*Blood Culture before antibiotics*	185(63.3)	111(52.8)	74(90.2)	***< 0.001***
*Broad Spectrum Antibiotics*	203(69.5)	131(62.3)	72(87.8)	***< 0.001***
Q19– Antibiotic use after diagnosis				
*1 h*	59(20.2)	31(14.7)	28(34.1)	***< 0.001***
Q20– Fluid Resuscitation Prior to ICU				
*True*	212(72.6)	139(66.1)	73(89.0)	***< 0.001***
Q21– Colloid solution preferable to crystalloid				
*False*	90(30.8)	51(24.2)	39(47.5)	***< 0.001***
Q22– Indications for Blood Culture				
*Chills*	94(32.1)	59(28.0)	35(42.6)	***0.017***
*Hypothermia*	78(26.7)	55(26.1)	23(28.0)	0.748
*Neutropenia*	121(41.4)	79(37.6)	42(51.2)	***0.034***
Q23– Indications prolonged antimicrobial use				
*Undrainable Infectious foci*	69(23.6)	55(26.1)	14(17.0)	0.100
*S. aureus bacteremia*	116(39.7)	71(33.8)	45(54.8)	***0.001***
*Neutropenia*	55(18.8)	44(20.9)	11(13.4)	0.140
*Simultaneous fungal infection*	39(13.3)	34(16.1)	5(6.1)	***0.023***
Q24– Typical Antimicrobial Duration 7–10 days				
*True*	120(41.0)	78(37.1)	42(51.2)	***0.028***
INCORRECT ANSWERS				
Q18– Immediate Resuscitation Measures				
*Blood Transfusion*	47(16.0)	28(13.3)	19(23.1)	***0.040***
Q19– Antibiotic use after diagnosis				
*20 min*	114(39.0)	80(38.0)	34(41.4)	0.597
*45 min*	13(4.4)	8(3.80)	5(6.09)	0.396
*35 h*	3(1.0)	3(1.42)	0(0)	0.279
*don’t know*	103(35.2)	88(41.9)	15(18.2)	***< 0.001***
Q20– Fluid Resuscitation Prior to ICU				
*False*	12(4.1)	11(5.23)	1(1.2)	0.121
*don’t know*	68(23.2)	60(28.5)	8(9.8)	***0.001***
Q21– Colloid solution preferable to crystalloid				
*True*	62(21.2)	35(16.6)	27(32.9)	***0.002***
*don’t know*	140(47.9)	124(59.0)	16(19.5)	***< 0.001***
Q22– Indications for Blood Culture				
*Neutrophil Right Shift*	91(31.1)	72(34.2)	19(23.1)	0.066
*Don’t Know*	77(26.3)	62(29.5)	15(18.2)	0.051
Q23– Indications prolonged antimicrobial use				
*Don’t know*	126(43.1)	91(43.3)	35(42.6)	0.921
Q24– Typical Antimicrobial Duration 7–10 days				
*False*	35(11.9)	31(14.7)	4(4.9)	***0.020***
*Don’t Know*	137(46.9)	101(48.0)	36(43.9)	0.520

ICU: intensive care unit, *S. aureus*: Staphylococcus aureus

### Assessment of attitudes toward Sepsis management

A vast majority of medical (98.6%) and nursing students (97.6%) agree that more training on sepsis is needed. Both groups of students also agreed that sepsis bundles should be implemented at their respective hospitals (91.9% and 100% respectively). While very few medical students (8.6%) and nursing students (3.7%) indicated that they were aware of the SSC guidelines, approximately half of all the students agreed that healthcare workers were very likely or somewhat likely to correctly identify patients at risk for sepsis. Nursing students were more likely to agree with this statement compared to medical students [67/82 (81.7%) vs. 87/210 (41.4%, *p* < 0.001].

### Correct and incorrect knowledge and practice scores

**Fig. 1:**
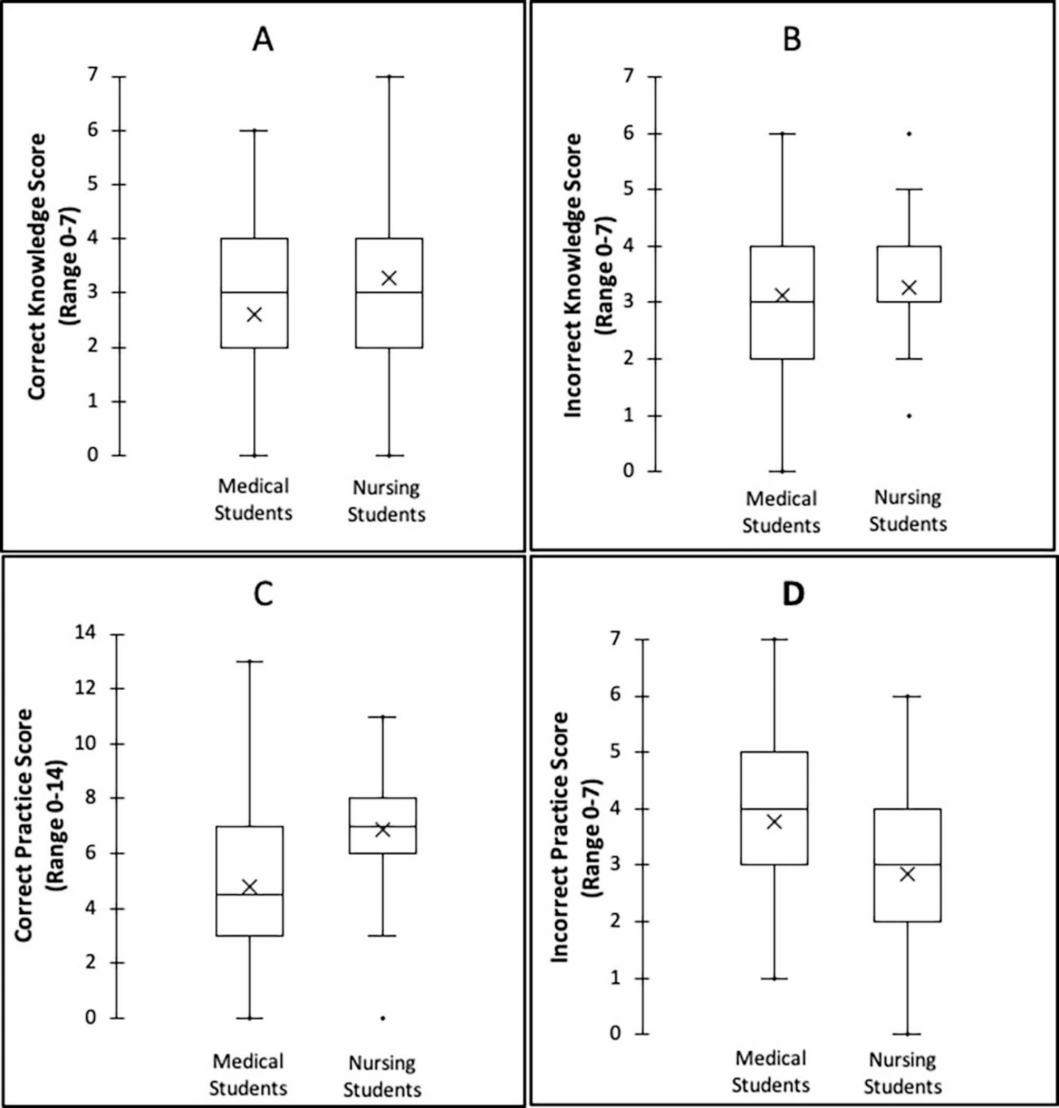
Mean ‘Correct’ and ‘Incorrect’ knowledge and practice scores among nursing and medical students. The distribution of scores among medical students is compared with that of nursing students. **A**. Correct Knowledge Score (Range 0–7), **B**. Incorrect Knowledge Score (Range 0–7), **C**. Correct Practice Score (Range 0–14), **D**. Incorrect Practice Score (Range 0–7)

Among all students, mean scores (±SD) for ‘correct knowledge’ (max 7), ‘incorrect knowledge’ (max 7), ‘correct practice’ (max 14) and ‘incorrect practice’ (max 7) were 2.8(±1.6), 3.2(±1.1), 5.4(±2.8) and 3.5(±1.4). Nursing students had higher mean ‘correct knowledge’ (3.3±1.7 vs. 2.6±1.5, *p* = 0.001) and ‘correct practice’ scores (6.9±2.2 vs 4.8±2.7, *p* < 0.001) compared to medical students. Correspondingly, nursing students had lower mean ‘incorrect practice’ scores (2.9±1.5 vs 3.8±1.3, *p* < 0.001), while there was no statistical difference in the incorrect knowledge scores between the 2 groups of students. (Fig. 1).

### Impact of current year of training on quantitative scores

**Fig. 2:**
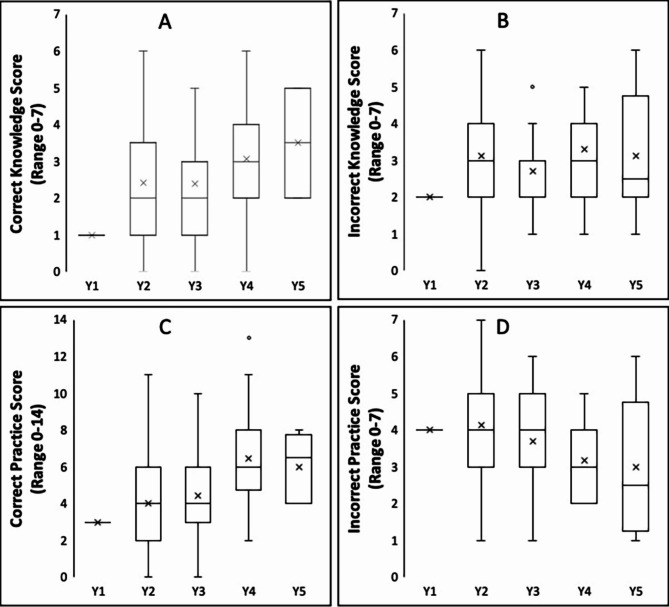
**A-D** The distribution of knowledge and practice scores among medical students. Scores are compared across years of medical school training (Y1-Y5). **A**: correct knowledge score, **B**: incorrect knowledge score, **C**: correct practice score, **D**: incorrect practice score

**Fig. 3:**
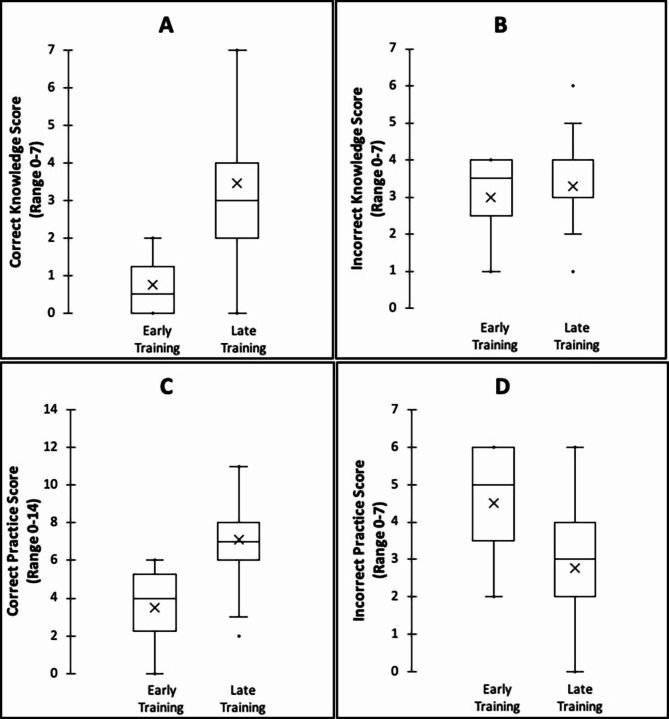
The distribution of knowledge and practice scores among nursing students. Comparison of scores among nursing students in early years of training (Y2/3) and those in final year of training (Y4 ). **A**: correct knowledge score, **B**: incorrect knowledge score, **C**: correct practice score, **D**: incorrect practice score

There were significant differences in correct and incorrect scores when compared across years of training. For medical students, a significant increase was noted for correct knowledge (1.0±0.0 to 3.5±1.3, *p* = 0.024) and correct practice (3.0±0.0 to 6.0±1.7, *p* < 0.001) scores, progressing from years 1 to 5 of training. Similarly, a significant decrease of incorrect practice scores (4.0±0.0 to 3.0±1.7, *p* < 0.001) was noted over progressive training years. There was no significant change in incorrect knowledge scores (Fig. 2A-D). For nursing students, a significant increase was noted for correct knowledge (1.4±1.5 to 3.4±1.6, *p* = 0.007) and correct practice (4.0±2.3 to 7.1±2.0, *p* = 0.001) scores from early to late years of training. There was no significant change in either incorrect knowledge or practice scores over the same period. (Fig. 3).

### Subgroup analysis– comparison of medical and nursing students in final stage of training

When comparing knowledge assessments of nursing and medical students in their final years of training, there were noted differences to the general comparison of students in all years. Notably, nursing students were no longer able to identify a low systolic blood pressure as an indication of sepsis more frequently than med students (67.6% vs. 67.2%, *p* = 0.971). Similarly, nursing students and medical student in their final years were equally likely to incorrectly identify an abnormal WBC as a sole indicator of sepsis (79.7% vs. 79.3%, *p* = 0.956). Interestingly, medical students were more likely to identify the correct definition of sepsis (Q11) in both the general and subgroup analysis but this difference achieved statistical significance in the subgroup analysis (69.0% vs. 47.3%, *p* = 0.013). Other differences between the 2 groups of students identified in the general analysis remained significant in the subgroup analysis but was generally less marked with noted improvements for medical students (Supplementary file S2).

When comparing practice assessments in the subgroup analysis, correct practices related to the need for broad spectrum antibiotic therapy were no longer identified more frequently by nursing versus medical students (90.5% vs. 82.8% *p* = 0.188). Similarly, the need for fluid resuscitation prior to transfer to the ICU (91.9% vs. 84.4%), *p* = 0.186), crystalloid solutions being more suitable for resuscitation than colloid (51.4% vs. 50.0%, *p* = 0.880), chills (44.6% vs. 39.7%, *p* = 0.572) and neutropenia (52.7% vs. 44.8%, *p* = 0.372) as an indication for blood culture and the typical antimicrobial duration of 7–10 days (50.0% vs. 37.9%), were no longer more frequently correctly identified by nursing compared to medical students. Interestingly, while nursing students were still able to more frequently identify the correct time of 1 h for antibiotic administration after diagnosis (36.5% vs. 19.0%, *p* = 0.028) and that *S. aureus* bacteremia is an indication for prolonged antimicrobial therapy (55.4% vs. 32.8%, *p* = 0.010), several improvements were noted for medical students compared to nursing students in the subgroup analysis. Specifically, this was noted with respect to identifying simultaneous fungal infections (27.6% vs. 6.8%, *p* = 0.001), undrainable infectious foci (43.1% vs. 17.6%, *p* = 0.001) and neutropenia (36.2% vs. 13.5%, *p* = 0.002) as indications for prolonged antimicrobial therapy in sepsis cases. (Supplementary file S2)

In the subgroup analysis, nursing students still had higher ‘correct practice’ scores (7.1±2.0 vs. 6.4±2.2, *p* = 0.041) than medical students but the difference was less marked. There was no longer a statistical difference in mean ‘correct knowledge’ scores (3.4±1.6 vs. 3.1±1.4, *p* = 0.193) or mean ‘incorrect practice’ scores (2.8±1.4 vs. 3.2±1.1, *p* = 0.126) when comparing nursing and medical students and there was still no statistical difference in the incorrect knowledge scores between the 2 groups of students. (Supplementary file, Figure S1).

### Sepsis training analysis

A total of 96 (32.8%) students indicated that at the time of taking the questionnaire, they had received some form of sepsis training. A majority of nursing students (55/82, 67.0%) had received sepsis training compared to only 41/210 (19.5%) medical students (*p* < 0.001).

As medical students progressed in their training, the proportion of participants who had received sepsis training increased from 0 to 28.6% by year 5. Similarly, 40% of nursing students in their early years of training indicated having sepsis training while 71.2% of nursing students in their final year of training indicated the same.

The impact of sepsis training was reflective of mean knowledge and practice scores obtained by students. Overall, students who had some form of sepsis training had higher mean correct knowledge (3.1±1.5 vs. 2.6±1.6, *p* = 0.011) and correct practice scores (6.8±2.5 vs. 4.6±2.6, *p* < 0.001) as well as lower mean incorrect practice scores (2.8±1.3 vs. 3.9±1.4, *p* < 0.001) compared to students who had no formal sepsis training. There was, however, no difference in mean incorrect knowledge scores. For medical students, those with sepsis training had higher mean correct practice scores (5.9±2.9 vs. 4.4±2.6, *p* = 0.002) as well as lower mean incorrect practice scores (3.2±1.2 vs. 3.9±1.3, *p* = 0.002). The same was true for nursing students, those with sepsis training had higher mean correct practice scores (7.4±1.9 vs. 5.8±2.3, *p* = 0.001) as well as lower mean incorrect practice scores (2.5±1.4 vs. 3.4±1.6, *p* = 0.012). Paradoxically, nursing students with sepsis training also had higher mean incorrect knowledge scores (3.5±0.9 vs. 2.9±1.1, *p* = 0.014).

## Discussion

This study is the first in the Caribbean evaluating healthcare students’ knowledge, practice and attitudes towards the management of sepsis. Our study indicates that a majority, usually just over half of students, could correctly identify most clinical signs of sepsis, including an altered mental state, hypotension and a dysregulated immune response to infection. In contrast, most students could not identify the signs that indicated the presence of septic shock such as high serum lactate and the need for vasopressors in management. Similarly, appropriate practices of sepsis management such as the need for broad spectrum antibiotics after blood culture collection and the need for fluid resuscitation prior to ICU admission, both in a timely manner, were identified as such by most students. However, while appropriate practice requires the use of crystalloid fluid for resuscitation and prolonged antimicrobial use in specific circumstances, most students could not correctly identify these specific practices as appropriate.

A majority of students were able to correctly identify signs of sepsis and to highlight the use of blood culture collection followed by broad-spectrum antibiotic therapy and fluid resuscitation as key components of sepsis management, indicating a good understanding of practice for the initial management of sepsis within the first 3 h. However, even for those aspects of sepsis management that most students could correctly identify, there were still usually 30–40% of students who were unable to do so. Further, most students did not have a clear understanding of the definition of septic shock and the appropriate management of such patients, which is concerning since these patients are at the greatest risk for mortality [1]. Recent graduates of healthcare institutions need to be competent in the recognition, assessment and management of sepsis to improve outcomes in septic patients [22, 25]. This study highlights the need for better sepsis education tools to prepare medical and nursing students in Jamaica, indicating almost all students agree that more formal sepsis training is needed and support implementation of sepsis bundles at their institutions.

In the general analysis, nursing students indicated correct sepsis management knowledge and practices more frequently than medical students that participated in this study with only a few instances in which the reverse was true. This difference was also highlighted in the quantitative scores in the 2 groups of students. Nursing students had higher overall mean correct knowledge and correct practice scores compared to medical students and lower incorrect practice scores, although there was no difference in incorrect knowledge scores between the 2 respondent groups. However, in our subgroup analysis comparing medical and nursing students in their final years of clinical training, we found notable differences to the general analysis in all students. While nursing students were still able to identify some elements of correct sepsis knowledge and practice, medical students showed significant improvement such that in many cases both groups of students identified correct responses with equal frequency. In several cases, particularly with respect to clinical indications for prolonged antibiotic administration and the correct definition for sepsis, medical students were more likely to identify the correct response than nursing students. This indicated that the general analysis was potentially impacted by the imbalance in years of training of captured medical students (tended to be earlier) and nursing students (tended to be later) and the differences in sepsis training curricula for the 2 groups of students. This subgroup analysis confirms some differences between the 2 groups and further highlights the impact of training on the improvements in sepsis knowledge and practice over their course of study.

We hypothesize that the noted difference between nursing and medical students may be due in large part to the more practical experience in direct patient interaction that the training of nurses emphasizes compared to the didactic focus of lectures in medical school training. Nursing students first experience direct patient interaction in their second year of training compared to the 3rd year of training for medical students in Jamaica. Since nursing has a particular focus on the practical applications of vital monitoring and drug administration over prolonged periods, it is likely that nursing students are often exposed to patients with sepsis over their training course. This gained practical experience provides a working knowledge of the common presentations of sepsis and the appropriate approach to its management, even in the absence of formal sepsis training. Indeed, one of the aspects of sepsis management that medical students were more likely to correctly identify was the need for prolonged antimicrobial administration in the case of sepsis with a simultaneous fungal infection; a relatively rare occurrence not likely to be experienced first-hand by many trainees.

Our study highlighted several variables that impacted quantitative scores representing appropriate and inappropriate sepsis knowledge and practice for both respondent groups. There was a significant increase in correct knowledge and practice scores in both nursing and medical students as they progressed from their early years of training to the final year of training. This is an expected finding in the study since presumably during the many years required to complete nursing and medical school training, students would periodically be exposed to several aspects of sepsis management. However, the expected decrease in incorrect knowledge and practice for sepsis management during progress in training was less consistently demonstrated. Furthermore, students indicating that they received formal sepsis training obtained higher correct knowledge and correct practice scores and lower incorrect practice scores. Interestingly, medical students reported that in year 1 none of them had yet received formal sepsis training but that increased to 28.6% of students in year 5, indicating that these sessions are not formalized as part of the training curriculum. In contrast, approximately 40% of nurses indicated receiving sepsis training during their early school years, the proportion increasing to just above 70% by their final year (year 4) of training. Overall, a larger proportion of nursing students, approximately two-thirds, had received formal sepsis training compared to close to 20% of all medical students. This is reflective of a more generally formalized inclusion of sepsis training as part of the nursing school curriculum and is also a likely contributor of the better performance in the questionnaire compared to medical students. This also highlights potential gaps in medical student training curricula that necessitate more formal sepsis training modules.

Unfortunately, we did not formally evaluate all the sources of sepsis information for respondents in our survey. Studies on graduate and undergraduate medical students in 2 medical schools in Australia on sepsis, were in line with other reports indicating that interns’ ability to recognize sepsis was poor. This study highlighted that medical students are underprepared with respect to the recognition and management of septic patients [21]. A multi-university study that evaluated final year nursing students’ exposure to education about sepsis indicated these students had limited knowledge in relation to recognizing, escalating and managing these patients [22]. Broad categories of health care students with variable levels of training have demonstrated inadequacy in recognizing key characteristics of sepsis presentation and management, highlighting a need for improved sepsis educational tools as a part of medical and nursing training programmes [21, 26].

Improving healthcare students’ understanding and awareness of sepsis may help to reduce sepsis global burden [27]. Education and training are essential components of this improvement in healthcare students [2] and systematic reviews have reported that active learning (stimulation and game-based learning) or blended learning (face to face and online learning simulation and game-based learning) were more successful than traditional didactic teaching in knowledge retention and perceived teaching methods [28]. The deficiencies identified by the questionnaires indicate that a review of current medical and nursing school curricula with respect to sepsis training should be undertaken by administrators. A recent consensus document on core sepsis-related competencies for medical students highlights essential competencies for low or lower-middle income countries like Jamaica [24]. These include core concepts of sepsis management such as definition of sepsis and septic shock and the urgency of antibiotic treatment and so this document can serve to guide development of sepsis curricula for healthcare students in Jamaica.

The strengths of this study include that it is the first study on sepsis education among Caribbean healthcare students and all participants could not consult a source of information prior to completion of the questionnaire and so likely accurately reflects the knowledge level of participant groups as they were observed. Use of an in-person rather than an online format facilitated a higher completion rate since there was no possibility for electronic deficits to affect data collection. Data was likely to be representative of students’ actual knowledge since communication while answering or a prior familiarity with the study questionnaire was not allowed for participants. However, there are several limitations, particularly the low response rate of both nursing (5.9%) and medical (13.7%) student participants in the study despite being open to all healthcare students, potentially a non-representative sample of students, and the use of the convenience sampling method which introduced selection bias. While the survey was open to all medical and nursing students at public institutions, some years of training were not captured and there was also an imbalance in exposure to training with more early years of medical students and more later years of nursing students captured. However, we completed a subgroup analysis of students in their late stage of training to get a more accurate representation of the comparison of nursing and medical students and to limit the impact of inherent structural differences in training curricula. We did not ask participants if they heard about sepsis before medical or nursing school.

## Conclusions

This survey showed that by the final year of their respective training courses, nursing and medical students demonstrated comparable performance with respect to knowledge and practice of sepsis in Jamaica. This indicates that effective sepsis medical education is important to improving population health as supported by the improved performance of both nursing and medical students who received either formal sepsis training or were in the late stage of training. The implementation of SSC care bundles during training should be considered as an educational tool for healthcare students in Jamaica.

## Electronic supplementary material

Below is the link to the electronic supplementary material.

Supplementary Material 1

Supplementary Material 2

## Data Availability

All data generated or analyzed during the current study are available from the corresponding author on reasonable request.

## Abbreviations

BCH: Bustamante Children Hospital
BP: Blood pressure
FMS: Faculty of Medical Sciences
ICUs: Intensive Care Units
KPH: Kingston Public Hospital
MAP: Mean Arterial Pressure
Q: Question
RR: Respiratory rate
SD: Standard deviation
SSC: Surviving Sepsis Campaign
STH: Spanish Town Hospital
Sys: Systolic
UHWI: The University Hospital of the West Indies
US: United States
USD: United States dollar
UWI: The University of the West Indies
WBC: White blood cell
Y: Year

## References

[1] Singer M, Deutschman CS, Seymour CW, Shankar-Hari M, Annane D, Bauer M, Bellomo R, Bernard GR, Chiche JD, Coopersmith CM, et al. The third international consensus definitions for sepsis and septic shock (Sepsis-3). JAMA. 2016;315(8):801–10. 26903338 10.1001/jama.2016.0287 PMC4968574

[2] Rudd KE, Johnson SC, Agesa KM, Shackelford KA, Tsoi D, Kievlan DR, Colombara DV, Ikuta KS, Kissoon N, Finfer S, et al. Global, regional, and National sepsis incidence and mortality, 1990–2017: analysis for the global burden of disease study. Lancet. 2020;395(10219):200–11. 31954465 10.1016/S0140-6736(19)32989-7 PMC6970225

[3] Fleischmann C, Scherag A, Adhikari NK, Hartog CS, Tsaganos T, Schlattmann P, Angus DC, Reinhart K. Assessment of global incidence and mortality of Hospital-treated sepsis. Current estimates and limitations. Am J Respir Crit Care Med. 2016;193(3):259–72. 26414292 10.1164/rccm.201504-0781OC

[4] Fleischmann-Struzek C, Mellhammar L, Rose N, Cassini A, Rudd KE, Schlattmann P, Allegranzi B, Reinhart K. Incidence and mortality of hospital- and ICU-treated sepsis: results from an updated and expanded systematic review and meta-analysis. Intensive Care Med. 2020;46(8):1552–62. 32572531 10.1007/s00134-020-06151-x PMC7381468

[5] Rhee C, Dantes R, Epstein L, Murphy DJ, Seymour CW, Iwashyna TJ, Kadri SS, Angus DC, Danner RL, Fiore AE, et al. Incidence and trends of sepsis in US hospitals using clinical vs claims data, 2009–2014. JAMA. 2017;318(13):1241–9. 28903154 10.1001/jama.2017.13836 PMC5710396

[6] Vincent JL, Sakr Y, Sprung CL, Ranieri VM, Reinhart K, Gerlach H, Moreno R, Carlet J, Le Gall JR, Payen D. Sepsis in European intensive care units: results of the SOAP study. Crit Care Med. 2006;34(2):344–53. 16424713 10.1097/01.ccm.0000194725.48928.3a

[7] Gerloni R, Mucci L, Casati C, Crociani A, Para O, Benetti E, Gnerre P, Bovero A, Romagnoli E, Tarquinio N, et al. Management of sepsis: from evidence to clinical practice. Italian J Med. 2016;10:308–28.

[8] Gauer RL. Early recognition and management of sepsis in adults: the first six hours. Am Fam Physician. 2013;88(1):44–53. 23939605

[9] Dellinger RP, Levy MM, Rhodes A, Annane D, Gerlach H, Opal SM, Sevransky JE, Sprung CL, Douglas IS, Jaeschke R, et al. Surviving sepsis campaign: international guidelines for management of severe sepsis and septic shock: 2012. Crit Care Med. 2013;41(2):580–637. 23353941 10.1097/CCM.0b013e31827e83af

[10] Evans L, Rhodes A, Alhazzani W, Antonelli M, Coopersmith CM, French C, Machado FR, McIntyre L, Ostermann M, Prescott HC et al. Surviving sepsis campaign: international guidelines for management of sepsis and septic shock 2021. Crit Care Med 2021, 49(11). 10.1097/CCM.0000000000005337 34605781

[11] Dellinger RP. Foreword. The future of sepsis performance improvement. Crit Care Med. 2015;43(9):1787–9. 26274702 10.1097/CCM.0000000000001231

[12] Levy MM, Dellinger RP, Townsend SR, Linde-Zwirble WT, Marshall JC, Bion J, Schorr C, Artigas A, Ramsay G, Beale R, et al. The surviving sepsis campaign: results of an international guideline-based performance improvement program targeting severe sepsis. Intensive Care Med. 2010;36(2):222–31. 20069275 10.1007/s00134-009-1738-3 PMC2826633

[13] Levy MM, Dellinger RP, Townsend SR, Linde-Zwirble WT, Marshall JC, Bion J, Schorr C, Artigas A, Ramsay G, Beale R, et al. The surviving sepsis campaign: results of an international guideline-based performance improvement program targeting severe sepsis. Crit Care Med. 2010;38(2):367–74. 20035219 10.1097/CCM.0b013e3181cb0cdc

[14] Levy MM, Rhodes A, Phillips GS, Townsend SR, Schorr CA, Beale R, Osborn T, Lemeshow S, Chiche JD, Artigas A, Dellinger RP. Surviving sepsis campaign: association between performance metrics and outcomes in a 7.5-year study. Intensive Care Med. 2014;40(11):1623–33. 25270221 10.1007/s00134-014-3496-0

[15] Kahn JM, Davis BS, Yabes JG, Chang CH, Chong DH, Hershey TB, Martsolf GR, Angus DC. Association between State-Mandated protocolized sepsis care and In-hospital mortality among adults with sepsis. JAMA. 2019;322(3):240–50. 31310298 10.1001/jama.2019.9021 PMC6635905

[16] Deis AS, Whiles BB, Brown AR, Satterwhite CL, Simpson SQ. Three-Hour bundle compliance and outcomes in patients with undiagnosed severe sepsis. Chest. 2018;153(1):39–45. 28987477 10.1016/j.chest.2017.09.031 PMC6689078

[17] Gabriella N, Anna E, Naomi T, Chiara Jane B, Pietro M: Physicians’ and nurses‘knowledge and attitudes in management of sepsis: An Italian study. Journal of Health and Social Sciences 2018, 3(1):13–26.

[18] Storozuk SA, MacLeod MLP, Freeman S, Banner D. A survey of sepsis knowledge among Canadian emergency department registered nurses. Australas Emerg Care. 2019;22(2):119–25. 31042531 10.1016/j.auec.2019.01.007

[19] Adegbite BR, Edoa JR, Rylance J, Jacob ST, Kawale P, Adegnika AA, Grobusch MP. Knowledge of health workers relating to sepsis awareness and management in Lambaréné, Gabon. Acta Trop. 2021;219:105914. 33831345 10.1016/j.actatropica.2021.105914

[20] Watkins RR, Haller N, Wayde M, Armitage KB. A multicenter survey of house staff knowledge about sepsis and the surviving sepsis campaign guidelines for management of severe sepsis and septic shock. J Intensive Care Med. 2020;35(2):187–90. 29088995 10.1177/0885066617737304

[21] Datta R, Di Tanna GL, Harley A, Schlapbach L, Nunnink L, Venkatesh B. An assessment of knowledge and education about sepsis among medical students: A multi-university survey. Crit Care Resusc. 2021;23:117–8. 38046385 10.51893/2021.1.RL2 PMC10692514

[22] Harley A, Massey D, Ullman AJ, Reid-Searl K, Schlapbach LJ, Takashima M, Venkatesh B, Datta R, Johnston ANB. Final year nursing student’s exposure to education and knowledge about sepsis: A multi-university study. Nurse Educ Today. 2021;97:104703. 33360011 10.1016/j.nedt.2020.104703

[23] Roye-Green K, Willis R, Priestley SR, Vickers I. Knowledge, practice and attitudes to the management of sepsis in Jamaica. J Crit Care Med (Targu Mures). 2022;8(4):232–41. 36474617 10.2478/jccm-2022-0024 PMC9682927

[24] Gomersall ELM, Ling L, Reinhart K, Bion V, Ekesh A, Adu-Takyi C, Azevedo LCP, Banguti PR, Cohen J, Diaz JV, et al. Core sepsis-related competencies for medical students: an international consensus by Delphi technique. BMC Med Educ. 2024;24(1):653. 38862952 10.1186/s12909-024-05525-9 PMC11167876

[25] Al Ansari M, Al Bshabshe A, Al Otair H, Layqah L, Al-Roqi A, Masuadi E, Alkharashi N, Baharoon S. Knowledge and confidence of Final-Year medical students regarding critical care Core-Concepts, a comparison between Problem-Based learning and a traditional curriculum. J Med Educ Curric Dev. 2021;8:2382120521999669. 35187261 10.1177/2382120521999669 PMC8855474

[26] Choy CL, Liaw SY, Goh EL, See KC, Chua WL. Impact of sepsis education for healthcare professionals and students on learning and patient outcomes: a systematic review. J Hosp Infect. 2022;122:84–95. 35045340 10.1016/j.jhin.2022.01.004

[27] Schlapbach LJ, Kissoon N, Alhawsawi A, Aljuaid MH, Daniels R, Gorordo-Delsol LA, Machado F, Malik I, Nsutebu EF, Finfer S, Reinhart K. World sepsis day: a global agenda to target a leading cause of morbidity and mortality. Am J Physiol Lung Cell Mol Physiol. 2020;319(3):L518–22. 32812788 10.1152/ajplung.00369.2020

[28] Liu Q, Peng W, Zhang F, Hu R, Li Y, Yan W. The effectiveness of blended learning in health professions: systematic review and Meta-Analysis. J Med Internet Res. 2016;18(1):e2. 26729058 10.2196/jmir.4807 PMC4717286

