# Supplementary material for: Knowledge, practice and attitudes of healthcare students to sepsis management in Jamaica
Source: BMC Med Educ. 2025 Apr 17;25:565. doi: 10.1186/s12909-025-07122-w (PMC12007360; doi:10.1186/s12909-025-07122-w)
Supplement: Supplementary file 1 — Supplementary Material 1 [file 12909_2025_7122_MOESM1_ESM.pdf]

# Sepsis Questionnaire

Dear Colleague,

The Microbiology Department University of the West Indies is aiming to decrease sepsis mortality and morbidity by improving sepsis awareness and care. Please complete the following questionnaire about your knowledge of sepsis and its treatment. This questionnaire is to find out information on sepsis awareness and practice. The results will help guide education and training on sepsis.

After completing the consent form please answer the following questions:

1. What is your position (Tick all that apply)?
   - ☐ Medical student (what year) ------
   - ☐ Student nurse (what year) -------
   - ☐ Nurse
   - ☐ Intern/SHO
   - ☐ Resident
   - ☐ Senior/Chief resident
   - ☐ GP
   - ☐ Consultant
   - ☐ Other---------------------

2. Where are you working?
   - ☐ Public hospital
   - ☐ Private hospital
   - ☐ Outpatient/ Clinic
   - ☐ Other--------------------

3. If hospital based, to which hospital are you employed?
   - ☐ UHWI
   - ☐ KPH
   - ☐ BCH
   - ☐ CRH
   - ☐ Other----------------------

4. In which parish (es) is /are you working?
   ----------------------------------

5. Experience (years post registration)
   - ☐ 0-5
   - ☐ 6-11
   - ☐ 12-17
   - ☐ 18-23
   - ☐ ≥24

6. Do you have postgraduate training?
   - ☐ Yes
   - ☐ No

7. If yes, state your specialty
   - ☐ Internal medicine
   - ☐ Emergency medicine
   - ☐ Anaesthesia/ Critical care
   - ☐ Paediatric
   - ☐ Surgery
   - ☐ Obstetrics and gynaecology
   - ☐ Other--------------------------

8. What is your gender?
   - ☐ Female
   - ☐ Male

9. Have you received any training on sepsis?
   - ☐ Yes
   - ☐ No

The following questions will help us to understand how well healthcare workers understand sepsis infection. Please remember this is not a test.

## KNOWLEDGE

10. Patients with infection are likely to be septic (by the quick Sequential (Sepsis-related) Organ Failure Assessment) with which of the following? ( Tick all that apply)
   ✓ Systolic blood pressure of 100 mm Hg or less (1 point – correct knowledge score)
   ✓ Altered mental status (1 point – correct knowledge score)
   ✓ Respiratory rate 22 breaths/min or greater (1 point – correct knowledge score)
   ☐ Hyperventilation $PaCO_2$ < 32 mm Hg (1 point – incorrect knowledge score)
   ☐ White blood cell count >12,000 cells/$mm^3$ or < 4, 000 cells/ $mm^3$ (1 point – incorrect knowledge score)
   ☐ Don't know

11. What is sepsis? (Tick all that apply)
   ✓ A dysregulated host response to infection. (1 point – correct knowledge score)
   ☐ Blood poisoning. (1 point – incorrect knowledge score)
   ☐ Presence of bacteria in the blood. (1 point – incorrect knowledge score)
   ☐ An allergic reaction. (1 point – incorrect knowledge score)
   ☐ Don't know
   .

12. What is septic shock? Sepsis with persisting hypotension despite adequate volume resuscitation plus (Tick all that apply)
   ✓ Requirement of vasopressors to maintain mean arterial pressure ≥65mm Hg (1 point – correct knowledge score)
   ✓ Serum lactate level >2mmol/L (1 point – correct knowledge score)
   ☐ Sepsis associated with cardiovascular dysfunction. (1 point – incorrect knowledge score)
   ☐ Don't know

13. The worldwide annual mortality rate of sepsis ranges from
   ☐ 1 to 5% . (1 point – incorrect knowledge score)* for any answer other than the correct one
   ☐ 10 to 15 %
   ☐ 20 to 30%

- ✓ 20 to 50 % (1 point – correct knowledge score)
- ☐ Don’t know

14. Have you heard about the Surviving Sepsis Campaign guidelines?
    - ☐ Yes
    - ☐ No

## ATTITUDE

15. Do you think you need more training on sepsis?
    - ☐ Yes
    - ☐ No

16. To what extent do you think healthcare workers can identify patients at great risk for sepsis?
    - ☐ Strongly agree
    - ☐ Somewhat agree
    - ☐ Neutral.
    - ☐ Somewhat disagree.
    - ☐ Strongly disagree.

17. Do you think sepsis bundles should be implemented at your hospital/ practice?
    - ☐ Yes
    - ☐ No

## PRACTICE

18. In the resuscitation of septic patients, the following is to be completed within 3 hours. (Tick all that apply)
    - ✓ Measure lactate level (1 point – correct practice score)
    - ✓ Obtain blood cultures prior to antibiotics administration (1 point – correct practice score)
    - ✓ Administration of broad-spectrum antibiotic (1 point – correct practice score)
    - ☐ Blood transfusion to correct hypotension. (1 point – incorrect practice score)
    - ☐ Don’t know

19. Antibiotics should be administered by ------------of the presumptive diagnosis of sepsis
    - ☐ 20 minutes (1 point – incorrect practice score)* for any answer other than the correct one
    - ☐ 45 minutes
    - ✓ 1 hour (1 point – correct practice score)

☐ 35 hours
☐ Don't know

20. Adequate fluid resuscitation should be started before ICU admission in sepsis
    ✓ True (1 point – correct practice score)
    ☐ False (1 point – incorrect practice score)* for any answer other than the correct one
    ☐ Don't know

21. Colloid solutions should be used in preference to crystalloid as part of fluid resuscitation in septic shock
    ☐ True (1 point – incorrect practice score)* for any answer other than the correct one
    ✓ False (1 point – correct practice score)
    ☐ Don't know

22. Which of the following is an indication for obtaining blood culture? (Tick all that apply)
    ✓ Chills (1 point – correct practice score)
    ✓ Hypothermia (1 point – correct practice score)
    ✓ Neutropenia (1 point – correct practice score)
    ☐ Right shift of neutrophils (1 point – incorrect practice score)* for any answer other than the correct ones
    ☐ Don't know

23. In septicaemia, longer duration of antimicrobial therapy is indicated in the following(Tick all that apply)
    ✓ Undrainable foci of infection (1 point – correct practice score)
    ✓ Bacteremia with *Staphylococcus aureus* (1 point – correct practice score)
    ✓ Neutropenia (1 point – correct practice score)
    ✓ Some fungal infection (1 point – correct practice score)
    ☐ Don't know (1 point – incorrect practice score)

24. In septicaemia, the duration of antimicrobial therapy is typically 7 to 10 days.
    ✓ True (1 point – correct practice score)

- ☐ False (1 point – incorrect practice score)* for any answer other than the correct one
- ☐ Don’t know

**THANK YOU FOR YOUR PARTICIPATION.**
