# Supplementary material for: Knowledge, practice and attitudes of healthcare students to sepsis management in Jamaica
Source: BMC Med Educ. 2025 Apr 17;25:565. doi: 10.1186/s12909-025-07122-w (PMC12007360; doi:10.1186/s12909-025-07122-w)

Table S1. Assessment of Sepsis Management Knowledge in Nursing and Medical Students in Final Years of Training – Subgroup Analysis (Q10-13)

Variable\Statistic	Med Student N=58 (%)	Nurse Student N=74 (%)	p-value
Q10 – Signs of Sepsis			
-Systolic BP of 100mmHg or less	***39(67.2)***	***50(67.6)***	***0.971†***
-Altered Mental State	33(56.9)	48(64.9)	*0.354*
-Respiratory rate >22 breaths/min	34(58.6)	37(50.0)	*0.327*
Q11 – Sepsis Definition			
- dysregulated host response to infection	***40(69.0)***	***35(47.3)***	***0.013****
Q12 – Septic Shock Definition			
-Hypotension &Vasopressors for MAP	24(41.4)	48(64.9)	0.007
- Hypotension & Serum Lactate>2mmol/L	10(17.2)	32(43.2)	0.002
Q13 – Sepsis Annual Mortality Rate			
- 20 to 50%	1(1.7)	5(6.8)	*0.172*
Q10 – Signs of Sepsis			
-PaCO2<32mmHg	21(36.2)	18(24.3)	*0.140*
-Abnormal WBC <4 or >12 x10^3 /ul	***46(79.3)***	***59(79.7)***	***0.956†***
Q11 – Sepsis Definition			
-Blood Poisoning	5(8.6)	8(10.8)	*0.680*
-Bacteremia	31(53.4)	60(81.1)	0.001
-Allergic Reaction	0(0)	1(1.4)	*0.384*
Q12 – Septic Shock Definition			
- Hypotension & cardiovascular dysfunction	30(51.7)	28(37.8)	*0.113*
Q13 – Sepsis Annual Mortality Rate			
- 1 to 5%	4(6.9)	1(1.3)	*0.100*
- 10-15%	***16(27.6)***	***9(12.1)***	***0.026****
- 20 to 30%	8(13.7)	12(16.2)	*0.704*
- don't know	29(50.0)	47(63.5)	*0.121*

MAP: mean arterial pressure, mmHg – millimeters mercury, PaCO2: arterial partial pressure carbon dioxide, WBC – white blood cell. ***Note that differences in the subgroup analysis compared to the general analysis in all students are italicized in bold. † : p-value no longer significant. * : p-value now achieved significance.***

Table S2. Assessment of Sepsis Management Practice in Nursing and Medical Students in Final Years of Training – Subgroup Analysis (Q18-24)

**Variable\Statistic**	**Med Student N=58 (%)**	**Nurse Student N=74 (%)**	**p-value**
CORRECT ANSWERS			
Q18 – Immediate Resuscitation Measures			
Measure Lactate	21(36.2)	56(75.7)	<0.001
Blood Culture before antibiotics	41(70.6)	70(94.6)	<0.001
Broad Spectrum Antibiotics	***48(82.8)***	***67(90.5)***	***0.188†***
Q19 – Antibiotic use after diagnosis			
1hr	11(19.0)	27(36.5)	0.028
Q20 – Fluid Resuscitation Prior to ICU			
True	***49(84.4)***	***68(91.9)***	***0.186†***
Q21 – Colloid solution preferable to crystalloid			
False	**29(50.0)**	**38(51.4)**	**0.880†**
Q22 – Indications for Blood Culture			
Chills	***23(39.7)***	***33(44.6)***	***0.572†***
Hypothermia	19(32.8)	21(28.4)	0.590
Neutropenia	***26(44.8)***	***39(52.7)***	***0.372†***
Q23 – Indications prolonged antimicrobial use			
Undrainable Infectious foci	***25(43.1)***	***13(17.6)***	***0.001****
S. aureus bacteremia	19(32.8)	41(55.4)	0.010
Neutropenia	***21(36.2)***	***10(13.5)***	***0.002****
Simultaneous fungal infection	16(27.6)	5(6.8)	0.001
Q24 – Typical Antimicrobial Duration 7-10 days			
True	***22(37.9)***	***37(50.0)***	***0.169†***
INCORRECT ANSWERS			
Q18 – Immediate Resuscitation Measures			
Blood Transfusion	7(12.1)	15(20.3)	***0.213†***
Q19 – Antibiotic use after diagnosis			
20min	27(46.6)	31(41.9)	0.596
45min	3(5.2)	4(5.4)	0.958
35hrs	2(3.4)	0(0)	0.111
don't know	***15(25.9)***	***12(16.2)***	***0.175†***
Q20 – Fluid Resuscitation Prior to ICU			
False	3(5.2)	1(1.3)	0.208
don't know	***6(10.3)***	***5(6.8)***	***0.464†***
Q21 – Colloid solution preferable to crystalloid			
True	***13(22.4)***	***24(32.4)***	***0.206†***
don't know	***16(27.6)***	***12(16.2)***	***0.115†***
Q22 – Indications for Blood Culture			
Neutrophil Right Shift	***29(50.0)***	***18(24.3)***	***0.002****
Don't Know	9(15.5)	14(18.9)	0.613
Q23 – Indications prolonged antimicrobial use			
Don't know	17(29.3)	31(41.8)	0.138
Q24 – Typical Antimicrobial Duration 7-10 days			
False	15(25.9)	4(5.4)	0.001
Don't Know	21(36.2)	33(44.6)	0.334

ICU: intensive care unit, *S. aureus*: Staphylococcus aureus. ***Note that differences in the subgroup analysis compared to the general analysis in all students are italicized in bold. † : p-value no longer significant. * : p-value now achieved significance.***

Figure S1. Mean 'Correct' and 'Incorrect' Knowledge and Practice Scores among Nursing and Medical Students in Final Years of Training
The distribution of scores among medical students (Y4 & Y5) are compared with that of nursing students (Y4) in their final stage of clinical training. A. Correct Knowledge Score (Range 0-7), B. Incorrect Knowledge Score (Range 0-7), C. Correct Practice Score (Range 0-14), D. Incorrect Practice Score (Range 0-7).

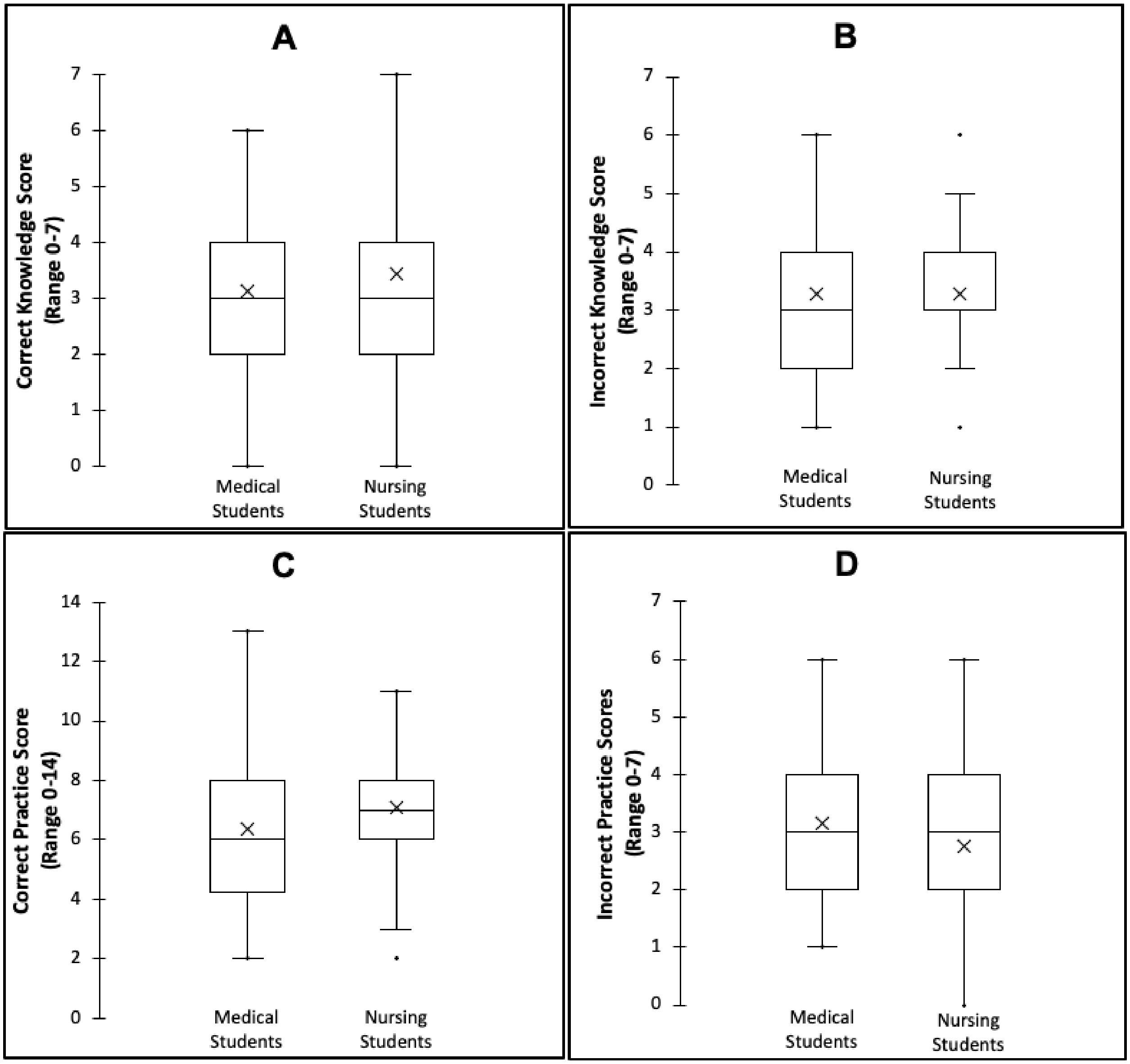

Supplement: Supplementary file 2 — Supplementary Material 2 [file 12909_2025_7122_MOESM2_ESM.pdf]
